# EARS-DM: Efficient Auto Correction Retrieval Scheme for Data Management in Edge Computing

**DOI:** 10.3390/s18113616

**Published:** 2018-10-24

**Authors:** Kai Fan, Jie Yin, Kuan Zhang, Hui Li, Yintang Yang

**Affiliations:** 1 State Key Laboratory of Integrated Service Networks, Xidian University, Xi’an 710071, China; yj06402@163.com (J.Y.); lihui@mail.xidian.edu.cn (H.L.); 2 Department of Electrical and Computer Engineering, University of Nebraska-Lincoln, NE, 68588 USA; kuan.zhang@unl.edu; 3 Key Laboratory of the Ministry of Education for Wide Band-Gap Semiconductor Materials and Devices, Xidian University, Xi’an 710071, China; ytyang@xidian.edu.cn

**Keywords:** edge computing, privacy, multi-keyword, automatic error correction, R-tree, relevance ranked

## Abstract

Edge computing is an extension of cloud computing that enables messages to be acquired and processed at low cost. Many terminal devices are being deployed in the edge network to sense and deal with the massive data. By migrating part of the computing tasks from the original cloud computing model to the edge device, the message is running on computing resources close to the data source. The edge computing model can effectively reduce the pressure on the cloud computing center and lower the network bandwidth consumption. However, the security and privacy issues in edge computing are worth noting. In this paper, we propose an efficient auto-correction retrieval scheme for data management in edge computing, named EARS-DM. With automatic error correction for the query keywords instead of similar words extension, EARS-DM can tolerate spelling mistakes and reduce the complexity of index storage space. By the combination of TF-IDF value of keywords and the syntactic weight of query keywords, keywords who are more important will obtain higher relevance scores. We construct an R-tree index building with the encrypted keywords and the children nodes of which are the encrypted identifier FID and Bloom filter BF of files who contain this keyword. The secure index will be uploaded to the edge computing and the search phrase will be performed by the edge computing which is close to the data source. Then EDs sort the matching encrypted file identifier FID by relevance scores and upload them to the cloud server (CS). Performance analysis with actual data indicated that our scheme is efficient and accurate.

## 1. Introduction

### 1.1. Cloud Computing and Edge Computing

Cloud is a metaphor for networks and the Internet. A cloud server is a simple, efficient, secure, and scalable computing service. Cloud computing is a pay-per-use model that provides usable, convenient, on-demand network access into a configurable pool of computing resources and provides unlimited storage capacity, lower computational costs and improved computing performance. However, the cloud server (CS) is not completely trustworthy. It may analyze and speculate with the user’s data to extract useful information. Therefore, the user’s privacy data needs to be encrypted before being uploaded to the CS.

On the other hand, the performance of cloud computing is limited by network latency, outages, and network bandwidth [1]. Cloud computing is generally accessed through a remote network and many time costs in the transmission and processing phrases. Moreover, the network speed depends on bandwidth, which is much lower than in a LAN. Once the network is interrupted, the service cannot be accessed. Although cloud centers have powerful processing capabilities, the challenge of transferring massive amounts of data to the cloud center appeared. Compared with traditional cloud computing, a faster, higher quality and lower cost services is available for nearby devices, which effectively solves the bottleneck of data transmission in cloud computing to a certain extent.

Edge computing, which extends cloud computing to the edge of the network, enables message to be acquired and processed at low prices. Many terminal devices are being deployed in the edge network to sense and deal with the massive data. By migrating part of the computing tasks from the original cloud computing model to the edge device, the message is running on the computing resources close to the data source. Edge computing model can effectively reduce the pressure of the cloud computing center and lower the network bandwidth consumption. In short, cloud computing is suitable for non-real-time, long-cycle data, business decision scenarios, and edge computing has an irreplaceable role in real-time, short-cycle data, local decision-making and other scenarios.

**Figure 1:**
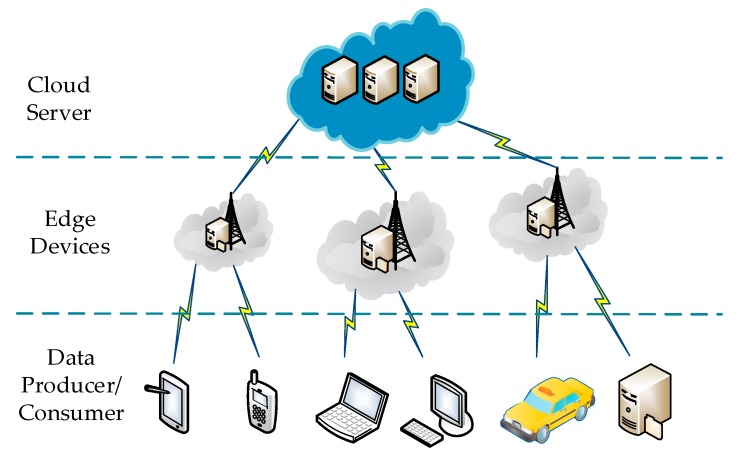
The structure of edge network.

We consider the edge network as a three-tiered architecture, as shown in Figure 1. Data producers/consumers are the devices who can be either data producers or data consumers. They contain a large number of sensors, control components and measuring components and communication components. These communication components may be separate or combined with other components. The edge devices mainly implement convergence and interconnection. In addition to network connectivity and management, the functions of edge devices also include edge computing, on-site processing and ensuring the survival of the business locally. In addition, protocol conversion is also an important function of this layer. The cloud server offers unified processing on the transmitted data and manages the lower layer by providing network deployment and configuring automation tools.

The system model in edge computing is different from that in traditional cloud computing. By using edge devices to provide users with networking computing and storage services, they only need less communication and computational costs. In the new EARS-DM scheme, we consider four different entities in the system model, including cloud server (CS), edge devices (EDs), data owners (DOs) and data users (DUs).

The security and privacy problems cannot be ignored in edge computing [2]. Since the EDs are located far away from the cloud computing center and cost much lower than the cloud computing, their information are more likely of lower quality and worse reliability [3]. Sensitive information has to be encrypted before uploaded to the EDs.

Traditional search technology is based on plaintext, that is, whether the keyword submitted by DOs or the data information given by data server is in plaintext, leading to a serious information leak. In order to solve this problem, there comes searchable encryption technology [4]. In the searchable encryption mode, the basic technology of cryptography is used to ensure the privacy information and personal security of users.

### 1.2. Our Contribution

In this paper, we propose an efficient, auto correction retrieval scheme for data management in edge computing. We take an automatic error correction for the query keywords instead of similar words extension, which can tolerate spelling mistakes as well as reduce the complexity of index storage space. By syntactic parsing and figuring up keyword weight of query keywords, our scheme dedicated to satisfying the user search experience. Our contributions can be summarized as follows:

1. We provide automatic error correction for query keywords instead of similar words extension, which can tolerate spelling mistakes as well as reduce the complexity of index storage space.
2. We adopt a special R-tree index. It is constructed by encrypted keywords and the children nodes of which are the encrypted identifier FID and Bloom filter BF of files who contain this keyword. The secure index will be uploaded to the edge computing and the search phrase will be performed by the edge computing which is close to the data source.
3. For the particularity of multi-keyword matching, we provide a two-step matching method. We first insert all the encrypted keywords into the R-tree and then perform keywords matching. If the match is successful, we will continue to match the Bloom filter of the corresponding file. The higher the match score, the more the file matches the query keywords.
4. We consider both the TF-IDF value of keywords and the syntactic weight KW of query keywords. DOs calculate TF value of keywords and insert them into the Bloom filter of index. DUs computes query keyword syntactic weight KW through the syntactic parser as well as IDF value in trapdoor generation phrase. The value of KW* IDF is inserted into the Bloom filter of trapdoor. By comparison of the inner product of the Bloom filter, EDs calculate the matching degree between this file and all search terms. According to the relevance scores, the top-K FID list is sent to the CS.
5. We present supported functions, security analysis and performance analysis of our retrieval scheme, and the result indicates that our scheme is efficient and accurate.

### 1.3. Organization

The remainder of this paper is organized as follows: we introduce some related works in Section 2. We introduce some preliminaries in Section 3. In Section 4, we present the overview of EARS-DM Scheme. In Section 5, we propose our framework of efficient auto correction retrieval scheme for data management in edge computing. Security and performance analysis is illustrates in Section 6. In the end, the conclusion is represented in Section 7.

## 2. Related Work

### 2.1. Edge Computing

In recent years, the study and application of edge computing has achieved great results. Wireless sensor networks (WSNs) have grown rapidly with the development of the Internet of Things. For example, to collect more sensory information and reduce latency, Liu et al. [5] proposed a hybrid unicast joint broadcast aggregation schedule scheme. For better energy efficiency and low stable rate of recall, Xu et al. [6] adopted memory storage and processing to lower power consumption and two TTLs were designed to cache edge data. Tao et al. [7] studied the energy efficiency and performance guarantees in mobile edge computing. Li et al. [8] combined edge-centered computing (ECC) and content-centric networking (CCN) to increase efficiency.

Vehicular ad-hoc networks are one of the main components of the Internet of Things (IoT) and edge computing. Through the on-board sensor, vehicle networks can sense the surrounding environment and obtain information such as roads, vehicle positions and obstacles based on the perception. In the low cost transmission system of vehicle network, the transportation capacity of the mobile vehicle can be better utilized and the “car sharing” can be realized [9]. With the popularity of smart homes, a large amount of heterogeneous data has been generated, which has brought enormous challenges to the management and application of data [10]. Li et al. [11] first introduced the deep learning of IoT to edge computing and designed a new unloading strategy optimized by edge computing. In performance evaluation, it is shown that edge computing can effectively optimize the performance of deep learning.

### 2.2. Searchable Encryption

To ensure privacy, searchable encryption for data management in edge computing is widely applied. The attacker cannot acquire or tamper with any private data even if he has the same authority as legitimate users.

Song et al. [12] first proposed a searchable encryption work that allowing users to scan the whole ciphertext through the single keyword to match the entire encrypted file. Therefore, we can know the existence and frequency of the keyword. However, scanning the entire encrypted file is a huge amount of computation. Goh et al. [13] proposed the concept of secure index, which uses the Bloom filter to generate the index of the file. Boneh et al. [14] proposed a public key encryption scheme, which was improved by the anonymous identity-based encryption scheme. Moreover, it can be widely used in the mail system. Park et al. [15] proposed the secure index search algorithm. However, these works only offered single-keyword search or conjunctive search. 

Subsequent works began to study multiple keywords search, but only exact keyword was supported. In 2007, Boneh et al. [16] proposed a general Hidden Vector System that supports comparison queries, general subset queries and arbitrary conjunctions. Cao et al. [17] presented a multi-keyword sort scheme. In search process, it constructs a vector for the index and the keyword. Moreover, it achieves the sorting of the search results by vector operations. 

Very recently, a few works that supporting fuzzy keyword search was proposed. In 2012, Li et al. [18] first proposed a technique supporting fuzzy search of keywords, but only single keywords. Wang et al. [19] provided formal security proofs for this scheme. Liu et al. [20] proposed a scheme based on dictionary, which greatly reduces the index size. However, only single fuzzy keyword search was supported in these works. Moreover, the fuzzy function of these works was relying on an expanding set of words. 

In 2016, Wang et al. [21] proposed a multi-keyword fuzzy search scheme with LSH technique. In 2016, Fu et al. [22] proposed an improved scheme based on the work of Wang et al. For the remove of the order dimension, the anagram is mapped to the same vector in the scheme. Moreover, he proposed a semantic central keyword search scheme, named CKSER [23] in 2017. CKSER provides central keyword extraction and semantic search by WordNet to find synonyms. In 2018, Ye et al. [24] proposed a search scheme with user access rights. If the data user has no access to the files, he will not be able to obtain the matching files. Miao et al. [25] supported attribute comparison by using 0 coding and 1 coding, and then realized the attribute-based multi-keyword search scheme. Although these programs are advanced, they still have limitations. Due to the diversity of search functions, the search keyword processing will be more complicated and local calculation of user will be increased. Moreover, the extension of keywords is so complex that leading to a larger local storage overhead.

To further improve the existing methods, fuzzy search schemes [18,19,20,21,22,23] has been brought into focus due to they can tolerance typing errors. Nevertheless, schemes of multi-keyword fuzzy search are inefficiency while keywords are spelled incorrectly. In our previous works, we implemented fuzzy search with n-gram and Bloom filter techniques, and presented a new sorting algorithm using a comprehensive sort score [26]. To improve index efficiency in vehicle network, we used a hybrid index structure where binary trees are embedded in a B+ tree. Once the values in the B+ tree matched successfully, we will continue to traverse the binary tree to obtain the search results [27]. To further support spelling mistakes and improve indexing efficiency, in this paper, we propose an efficient auto correction retrieval scheme for data management in edge computing, named EARS-DM. With automatic error correction for the query keywords instead of similar words extension, EARS-DM can tolerate spelling mistakes and reduce the complexity of index storage space. By the combination of TF-IDF value of keywords and the syntactic weight of query keywords, keywords who are more important will obtain higher relevance scores. We construct an R-tree index building with the encrypted keywords and the children nodes of which are the encrypted identifier FID and Bloom filter BF of files who contain this keyword. The secure index will be uploaded to the edge computing and the search phrase will be performed by the edge computing which is close to the data source. Then EDs sort the matching encrypted file identifier FID by relevance scores and upload them to the cloud server (CS). Performance analysis with actual data indicated that our scheme is efficient and accurate.

## 3. Preliminaries

In this section, brief descriptions of TF-IDF, Bloom Filter and R tree are given as follows.

### 3.1. TF-IDF 

To evaluate the importance of a keyword in a set of files, we utilize TF-IDF rules [28] where the TF means the frequency of a given keyword within a file and the IDF is a measure of the general importance of a word in the entire file set. If a keyword appears many times in a file and appears few times in other files, the more it can represent the file. If the number of documents containing the keyword is smaller, the IDF is larger and has the better class distinguishing ability. Firstly, DOs extract the keyword set of $\left\{w_{1},w_{2},\ldots ,w_{m}\right\}$ from each file. For each keyword $w_{i}\in \{w_{1},w_{2},\ldots ,w_{m}\}$, the frequency is denoted as $f_{i}\in \{f_{1},f_{2},\ldots ,f_{m}\}$ and $f_{W}$ refers to frequency of total keywords. TF can be expressed as $TF\left(w_{i}\right)=\frac{f_{i}}{f_{W}}$ which are sent to the edge device. In the trapdoor generation phrase, $g_{i}$ represents the number of files containing the search keyword $w_{i}$ in the edge device. The value of IDF can be displayed as $IDF\left(w_{i}\right)=\log\left(\frac{D}{g_{i}+1}\right)$ where D is the total number of files in the edge device. In the search phrase, edge device can calculate the relevance score between the extracted keywords and the files by $SC\left(w_{i}\right)=TF\times IDF=\frac{f_{i}}{f_{W}}\cdot \left(\log\left(\frac{D}{g_{i}+1}\right)\right)$.

### 3.2. Bloom Filter

A Bloom filter [29] is actually a long binary vector with a series of random mapping functions, which can retrieve whether an element is in a set. A Bloom Filter consists of an array of m-bits, which is initialized by all 0 s.

**Figure 2:**
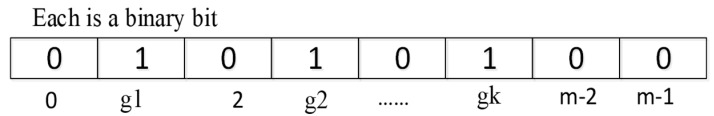
The working principle of Bloom filter.

For each element in array is mapped to values of $\left\{g_{1},g_{2},\;\ldots ,g_{k}\right\}$ by the independent hash functions of $\left\{f_{1},f_{2},\;\ldots ,f_{k}\right\}$. The positions of $\;\left\{Array\left[g_{1}\right],\;Array\left[g_{2}\right],\ldots ,\;Array\left[g_{k}\right]\right\}$ in the Bloom filter are set to 1. The working principle of Bloom filter is as shown in Figure 2.

### 3.3. R-Tree Data Structure

That R-tree can be used to speed up the nearest neighbor searching just meet the demand of our scheme. R tree solved the high-dimensional space search problem as well. It extends the idea of B-tree to multi-dimensional space, adopts the idea of dividing the space of B-tree, and adopts the method of merging and decomposing nodes when adding and deleting operations to ensure the balance of the tree. Therefore, the R-tree is a balanced tree used to store high-dimensional data.

**Figure 3:**
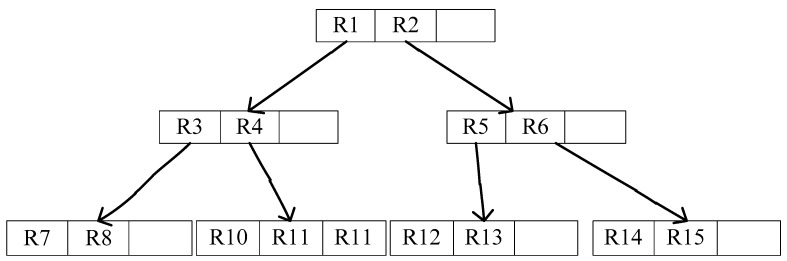
R-tree data structure.

The R-tree is a balanced tree extended from B-tree in the high-dimensional space [30]. Adopting the idea of space partition of the B-tree, the R-tree uses the method of merging and decomposing the nodes during the operations of adding and deleting to guarantee the balance of tree. Every leaf node of an R tree contains multiple pointers to different data, which can be either stored on the hard disk or in memory. The R-tree data structure is shown in Figure 3. Based on this data structure, when a high-dimensional space query is required, we only need to traverse the pointers among a few leaf nodes, which allow us to obtain answers without traversing all the data. Therefore, efficiency is significantly improved.

R-tree developed space partition by the means of the Minimal Bounding Rectangle (MBR) method, which uses rectangles to frame the space from the leaf nodes. The higher up the nodes, the greater the space framed. The root node stores the two largest rectangles, which frame all the remaining rectangles and all the data as well. The next level of nodes stores the next largest rectangle while leaf nodes stored the smallest rectangle. The data structure of leaf nodes is in the form of (M, tuple-id) while in non-leaf nodes is (M, child-pointer).

When querying for specific data, we start from the root node and select the corresponding first-level node, second-level node until the leaf nodes storing the smallest rectangular. Traversing all the pointers in the nodes, we check if it meets our requirements.

## 4. Problem Description

In this section, we briefly introduce the notations, system model and design goals of EARS-DM scheme.

### 4.1. Notations

The notations and descriptions used in this paper are listed in Table 1.

### 4.2. System Model

**Figure 4:**
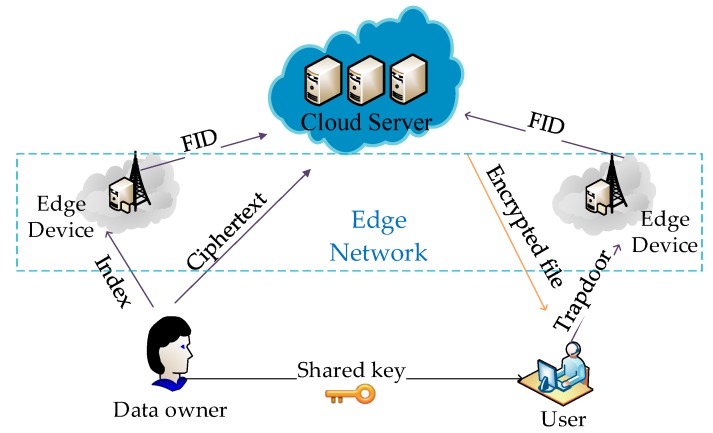
System model of retrieval construction in Edge computing system.

As introduced in Section 1, we consider four different entities in the system model, including data owners (DOs), cloud server (CS), edge devices (EDs) and authorized data users (DUs), as shown in Figure 4.

DOs are data producers who can also be the data users. DOs first extract keywords from files and calculate the TF weight for each keyword. DOs will build a security index *I* from keyword set $W=\{w_{1},w_{2},\ldots ,w_{m}\}$. Each keyword is followed by the associated FID. For each file, there is a Bloom filter that records the TF value of all the keywords in the file. Finally, the index *I* that contains the FID and TF value were uploaded to the edge device. The file identification ID is encrypted as FID and plaintext files are encrypted as C by a private key. Afterwards, the ciphertext files C and encrypted identification FID are uploaded to the cloud sever. In this system, DOs share with the authorized DUs private key and trapdoor generation key.

DUs are data consumers who can also be the data owners. To retrieve the desired ciphertext by query *Q*, DUs generate an associated trapdoor and calculate IDF value for each keyword and then uploads it to the edge device.

EDs are semi-trusted entities, which are responsible for the matching of the index and the trapdoor. Then they obtain some of the well-matched file FIDs and upload them to the CS.

The edge network solves the network latency problem of cloud computing. Edge device is very close to the data user that the data can reach the calculation and storage center of the edge computing only after one or a few hops, and the data will be directly calculated on the edge device without uploading to the cloud computing center. Edge computing leverages a large number of smart devices at the edge of the network. Although the resources of a single device are limited, a large number of devices can be organized to play a huge role.

CS is also a semi-trusted entity that provides data storage service in the system. After receiving the FID of files, CS sends the corresponding encrypted file to DUs. 

In the EARS-DM scheme, we assume that EDs and CS to be semi-trusted, namely “honest but curious”. Firstly, CS and EDs ensure the security of the stored information, that is, no tampering, addition or deletion of the users’ stored data. Second, CS and EDs are “honest” and follow the designed protocol to complete the assigned work. Nonetheless, curious analysis and inference may be from CS and EDs during the work.

### 4.3. Design Goals

1. *Automatic correction***.** We aim to take an automatic error correction for the query keywords which can tolerate spelling mistakes.
2. *Efficient indexing structure***.** We aim to adopt an index structure that can balance search efficiency with update operations. In this paper, the index structure is a R-tree structure, which is linking to Bloom filter.
3. *Consideration of the syntactic significance of each query keyword***.** Because the significance of different keywords with different types is distinct, we consider obtaining keyword weight KW through the syntactic parser.
4. *Relevance ranking with all search terms.* In the system, CS calculates the matching degree between this file and all search terms. According to the relevance scores, the importance of file is determined by the matching rate and sort from the top.

## 5. EARS-DM: Efficient Auto Correction Retrieval Scheme for Data Management

In this section, we first describe some fundamental algorithm of our scheme. Then we display the main framework of this paper.

### 5.1. Syntax Parser

Parsers based on Neural Networks [31] is widely used in NPL systems, which can better locate the relationship between modified adverbs and evaluation objects. By analyzing the syntactic structure by Stanford Parser, a sentence is described as a dependency pair as (word 1—number 1, word 2—number 2) between multiple tokens and word modification. As shown in Table 2. For each $q\in \{q_{1},q_{2},\ldots ,q_{k}\}$, the keyword weight of *q* is $KW(q)=\frac{k+k\sum R}{\sum_{i=1}^{k}\left(1+\sum R\right)}$. *R* is the significance in the dependency tree between the query keywords. 

### 5.2. Spelling Error Correction

**Figure 5:**
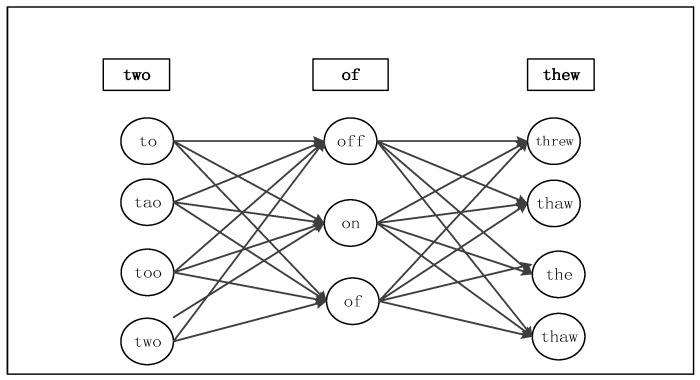
Noisy channel model.

When the quality of the query is low or even incorrect, the error should be firstly corrected and compensated, otherwise, the result will be disappointed. Spelling Correction is often used in word processing software, input method and search engine. According to the error types, we have Non-word Errors and Real-word Errors. In the model, the original word is converted into the noisy word through the noisy channel and decoded into the guessed word. The noisy channel model is shown in Figure 5.

Real-Word Spelling Correction. Kukich [32] has pointed out that 25%–40% of spelling mistakes belong to the Real-word type. Compared with the Non-word type, Real-word error correction is more difficult because each word in the sentence is treated as an object to be corrected. In general, the solution is in two steps. First, for each word in the phrase or sentence, the system generates the candidate set containing the words themselves, all single-letter edits English words and homophones. Second, the system chooses the best candidates through the noisy channel model and task specific classifier.

For example, a given sentence $S=\{w_{1},w_{2},\ldots ,w_{n}\}$, the candidate set for each $w_{i}$ is as below:

$$\begin{array}{l} Candidate\left(w_{1}\right)=\left\{w_{1},\;{w^{\prime }}_{1},\;{w^{\prime \prime }}_{1},\;{w^{\prime \prime \prime }}_{1},\ldots \right\}\\ Candidate\left(w_{2}\right)=\left\{w_{2},\;{w^{\prime }}_{2},\;{w^{\prime \prime }}_{2},\;{w^{\prime \prime \prime }}_{2},\ldots \right\}\\ \ldots \;\ldots \\ Candidate\left(w_{n}\right)=\left\{w_{n},\;{w^{\prime }}_{n},\;{w^{\prime \prime }}_{n},\;{w^{\prime \prime \prime }}_{n}\;,\ldots \right\} \end{array}$$

**Figure 6:**
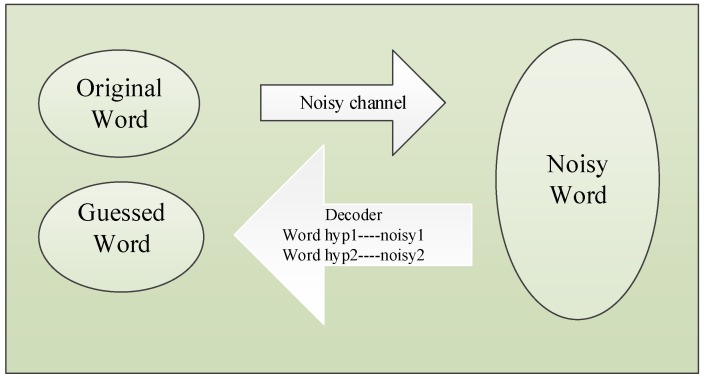
Real-word spelling correction model.

Finally, the sequence W with the highest probability is selected as an automatically-corrected sentence, which can be expressed into the word grid form and converted to HMM decoding process, as shown in Figure 6. There is a threshold in the system that controls the accuracy of the correction. 

### 5.3. Algorithms of EARS-DM

Definition 1 (EARS-DM: Efficient, Auto Correction Retrieval Scheme for Data Management in Edge computing).

**Setup (**$1^{\lambda }$**)**: {GP,UID}← Setup ($1^{\lambda }$). The setup algorithm is run by CA (Certificate Authority). CA is the organization responsible for issuing certificates to users and managing user certificates. In this algorithm, $\lambda_{}$ is the system input parameter and GP is the global parameter of the output. Let $G_{1},G_{2},G_{T}$ are both p-order multiplicative groups and p is a prime number. $g_{1}$ and $g_{2}$ are generators of $G_{1}$ and $G_{2}$, respectively. There is a bilinear map between $G_{1},G_{2}$ and $G_{T}$: $e:G_{1}\times G_{2}\to G_{T}$ and there are hash functions $G:G_{T}\to {\mathbb{Z}}_{p}$ and $H:{\left\{0,1\right\}}^{*}\to {\mathbb{Z}}_{p}$. CA randomly selects $a\in {\mathbb{Z}}_{p}$ and outputs $GP=\{p,G_{1},G_{2},G_{T},e,g_{1},g_{2},g_{2}^{a},H,G\}$. Users need to register with CA. CA then assigns each legitimate user a unique identity identifier UID. 

Key Generation (GP):

- Key Generation for CS: $\left(PK_{CS},SK_{CS})\leftarrow KeyGen_{CS}(GP\right)$. Key generation center inputs the public parameter GP and generates the public and private key pair $\left(PK_{CS},SK_{CS}\right)$ for CS.
- Key Generation for EDs: $\left(PK_{ED},SK_{ED})\leftarrow KeyGen_{ED}(GP\right)$. Key generation center inputs the public parameter GP and generates the public and private key pair $\left(PK_{ED},SK_{ED}\right)$ for EDs.
- Key Generation for DO: $\left((PK_{O},SK_{O}),K)\leftarrow KeyGen_{DO}(GP\right)$. Key generation center inputs the public parameter GP and randomly selects $u\in {\mathbb{Z}}_{q}^{*},Q\in G_{1}$ and computes $U=ug_{1}$. The public key and private key of DO is $\left\{PK_{O}=(GP,Q,U),SK_{O}=(GP,u)\right\}$. In addition, r random numbers $k_{1},\;k_{2},\ldots ,k_{r}$ as the input key of hash function are generated. The trapdoor key is $K=\{SK,\;k_{1},\;k_{2},\ldots ,k_{r}\}$.

**Index:**(I)←Index(W,TF,FID,K). On input the keyword set W, TF values, encrypted file identifier FID and the secret key K, the algorithm build an index tree I. Then the index tree I is encrypted by the public key $PK_{ED}$ of edge device and uploaded to the edge device.

**Trapdoor:**$(T_{Q})\leftarrow Trapdoor\left(IDF,Q,K\right)$. On input the query keyword set $Q=\{q_{1},q_{2},\ldots ,q_{k}\}$ IDF values and the secret key K, the algorithm generates the trapdoor $T_{Q}$. Then the trapdoor $T_{Q}$ is also encrypted by the public key $PK_{ED}$ of edge device and uploaded to the edge device.

**Search:**(FID)←Search(I,TQ). On receiving the trapdoor from DU, ED is to match the index I and trapdoor $T_{Q}$ to obtain the corresponding FID of files. The result of FID is uploaded to the CS and CS finds the correspondingly encrypted files and returns them to the DU. DU obtains the required encrypted files and decrypts them.

### 5.4. Our Framework

**Figure 7:**
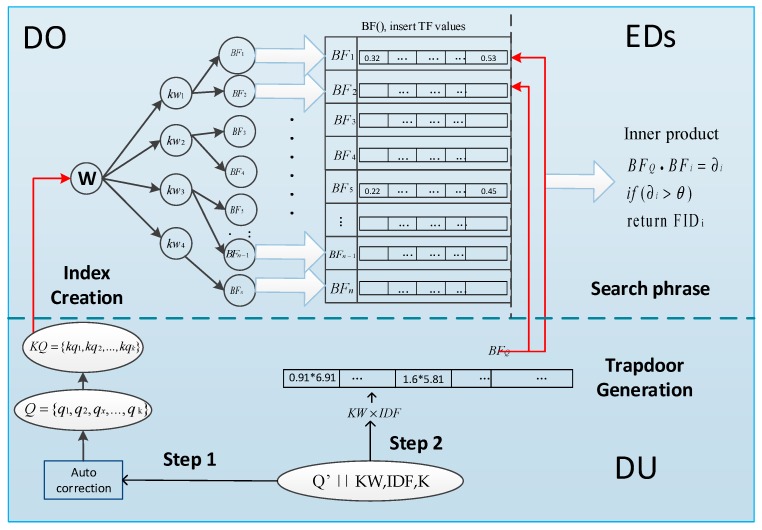
The main framework of our scheme.

The main steps of our framework including files encryption, index generation, trapdoor generation and search phrased, which is shown in Figure 7. Detailed introductions are as follows:

1. File encryption. DOs first extract the keyword set W from file set *F*. Next, DOs encrypt the identifier of file set *F* as FID and encrypt the file set $F=\{f_{1},\;f_{2},\ldots ,\;f_{n}\}$ as $C=\{c_{1},\;c_{2},\ldots ,\;c_{n}\}$. In the end, DOs upload the encrypted file set C and corresponding FID to the CS. 

2. Index Creation. In this phrase, DOs compute TF value for each keyword $w_{i}\in W$ and the TF value is mapped into the Bloom filter by the secret key K. The keyword set *W* are encrypted as tags $\left\{kw_{1},\;kw_{2},\ldots ,\;kw_{m}\right\}$, which are inserted into the R-tree. The children of $kw_{i}$ are the encrypted identifier FID and Bloom filter BF of files who contain this keyword $w_{i}$. Each file $f_{i}$ is corresponding to a Bloom filter $BF_{i}$ and an encrypted identifier $FID_{i}$. 

3. Trapdoor Generation. Initially, DUs make an error correction for the original keyword set $Q^{\prime }$. After the introduction of spelling auto correction for keywords, if DUs misspells some query keywords, the system will auto correct them to be the most similar keywords Q = $\left\{q_{1},q_{2},\ldots ,q_{k}\right\}$.

After correction, DUs calculate IDF value and keyword weight KW for each keyword q∈Q. The keyword set Q = $\left\{q_{1},q_{2},\ldots ,q_{k}\right\}$ are encrypted into tags KQ = $\left\{kq_{1},kq_{2},\ldots ,kq_{k}\right\}$, which are used to match the keyword tags of index. The value of IDF*KW is mapped into the Bloom filter of trapdoor by the secret key K. 

4. Search Phrase. According to the index structure, EDs first perform tags matching between index and trapdoor. If a tag matches, EDs continue to traverse its children to find all FID and BF of files containing this keyword. The Bloom filter BF of index is storing TF value while Bloom filter of trapdoor is storing the value of IDF*KW. EDs then make an inner product operation between the Bloom filter of index and trapdoor. The inner product results are the relevant scores $SC(w_{i})$, meaning the matching degree between this file and all search terms. Let θ be the threshold, if the relevant score is less than the threshold, the corresponding FID is discarded. Otherwise, the corresponding FID is order by the relevant score. Finally, the result of FID is uploaded to the CS and CS finds the corresponding encrypted files and returns them to the DU. DU obtains the required files and decrypts them. 

## 6. Analysis of Proposed Scheme

In this section, we describe the comparison of supported functions, security analysis and performance analysis of our retrieval scheme of data management.

### 6.1. Supported Functions

We compare the supported functionality by existing schemes and our scheme, as illustrated in Table 3. 

As we know, MRSE, a multi-keyword ranking scheme for encrypting cloud data was first proposed by Cao. Wang et al. [33] attempted to semantic extension and study the relevance degree for keywords. Later, Fu et al. proposed a semantic extended scheme, which is based on central Keywords. Chen et al. [34] tried to study multi-keyword fuzzy search with efficient index structure on encrypted cloud data. As we can see from Table 3, our scheme is multifunctional which stands by auto correction to tolerate spelling mistakes compared with other schemes. 

### 6.2. Security Analysis

*Data privacy*. To ensure that adversary is impossible to get any sensitive information from the ciphertext retrieval, the file and the keyword must be encrypted before upload to the CS. As long as the key is secure, the ciphertext is safe.

*Index and Trapdoor Privacy.* In our scheme, the index and trapdoor are both encrypted by secret key SK. Attacker cannot obtain the plaintext from the secure index or trapdoor without the trapdoor generation key.

*Trapdoor Unlinkability*. If the same keywords generate the same trapdoor, CS may perform a frequency guessing attack. Therefore, we introduce random numbers in the trapdoor generation process, so that even the same keywords will generate different trapdoors. Therefore, the cloud server cannot infer the relationship between the given trapdoors and meet the requirements for protecting the privacy of the keyword.

*Access control.* An untrusted server cannot search without authentication by a legitimate user.

### 6.3. Performance Analysis

In this section, we discuss the performance of our scheme. We implement prototypes of our scheme compared with CKSER and EliMFS. CKSER is a central keyword semantic extension ranking scheme while EliMFS is a multi-keyword fuzzy search scheme based on Gram Counting Order. CKSER-1 is the scheme based on known ciphertext model while CKSER-2 is based on known background model. We randomly selected 8000 files from Baidu Library and extracted about 15,500 distinct keywords. We used services of Tencent Cloud and used Cloud Virtual Machine (CVM) to simulate edge devices. We built 50 CVMs. The program is implemented with Python language on Ubuntu 16.04 sever with Intel^®^ Core (TM) i3-3240 CPU @ 3.40 GHz with 8.00 GB RAM.

*Index Creation***.** In this phrase, the main step is building the R-tree with tags and leaf nodes with Bloom filters BF and encrypted file identifier FID. Ideally, the insertion efficiency and search efficiency of the Bloom filter can be regarded as a constant, that is, O(1). The number of encrypted keywords in R tree depends mainly on the size of the keyword dictionary. Figure 7 illustrates that the time consumption of index creation grows linear trend with the number of keywords in dictionary as well as the number of files in file set. 

**Figure 8:**
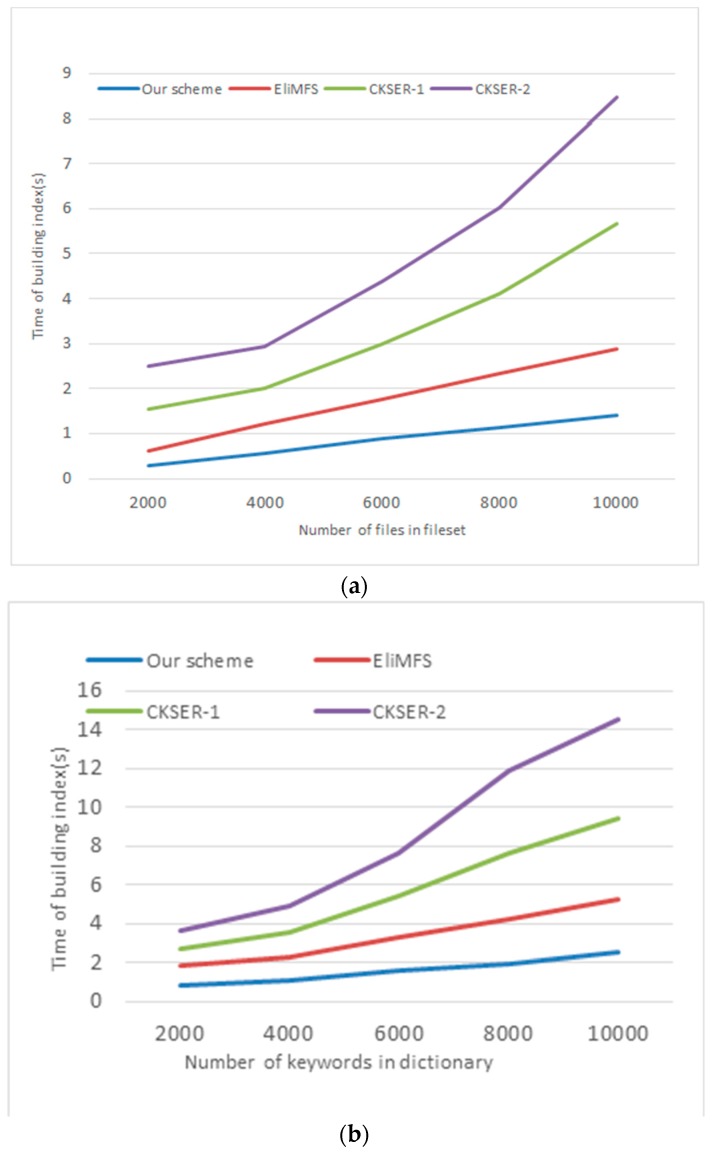
Time consumption of index creation. (**a**) Time consumption of building index changes with the file number of fileset; (**b**) Time consumption of building index changes with the keyword number of dictionary.

Figure 8a,b shows the time consumption of index creation. Figure 8a illustrates the time consumption changes with the files number in dataset, while size of the dictionary is *m* = 5000. Figure 8b illustrates the time consumption changes with the number of keywords in dictionary, while the files number is *n* = 3000.

**Figure 9:**
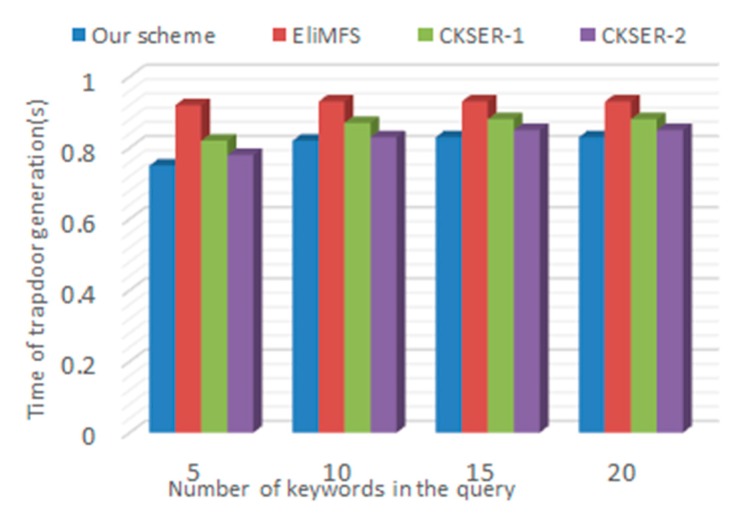
Time of trapdoor Generation.

**Figure 10:**
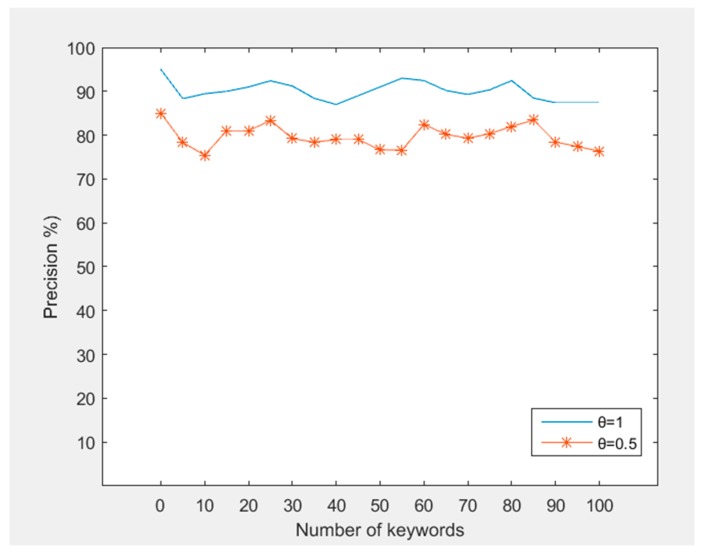
Precision of auto correction.

*Trapdoor Generation.* There are some main steps in trapdoor generation: (1) auto correction; (2) calculation of IDF and KW value; (3) building trapdoor. The time consumer of trapdoor depends mainly on the size of the query keywords. Figure 9 shows the trapdoor generation time slowly increasing with the number of query keywords. Due to the small number of query keywords, the processing time of (1) and (2) can be regarded as a constant. The insertion efficiency of the Bloom filter can be regarded as O (1). Figure 10 shows the precision of auto correction system changing with the number of keyword with different threshold. We can see that the points are evenly distributed around the average expected value. Precision depends on many factors, especially the proximity between the misspelled word and the target word.

**Figure 11:**
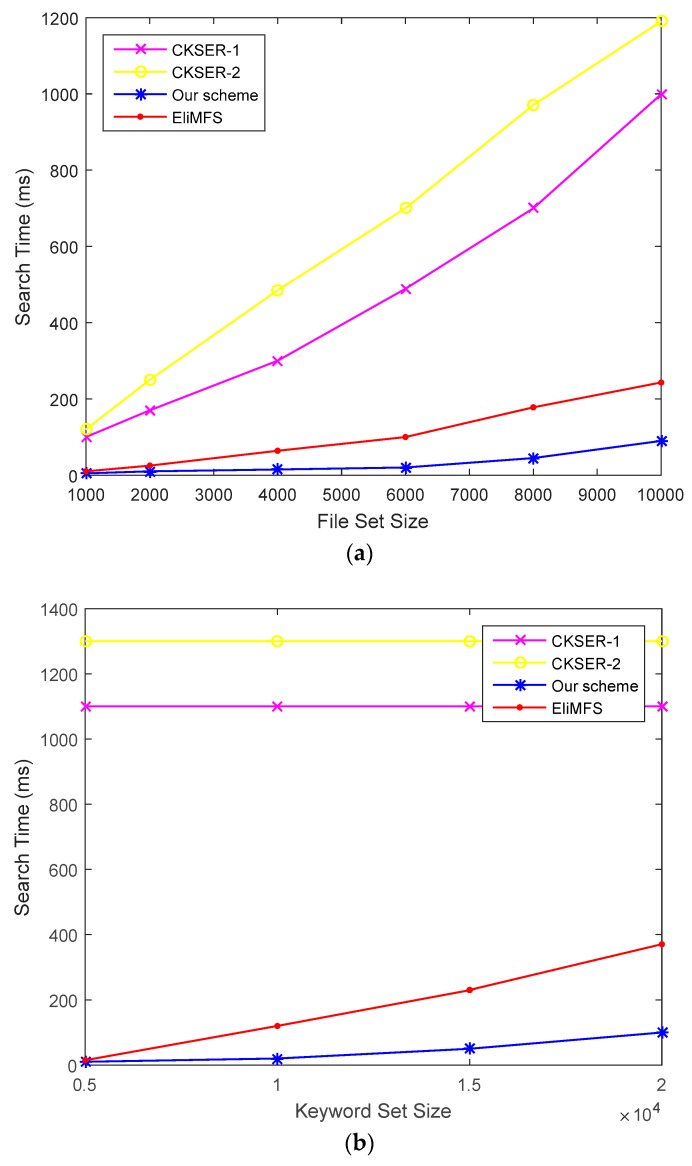
Time consumption of search process. (**a**) Time consumption of search process changes with the size of file set (*n* = 8000); (**b**) Time consumption of search process changes with the size of file set (*n* = 8000).

*Search Phrase.* The main step of search phrase includes keywords matching and the inner product computation between the index and the trapdoor. The search time of our schemes mainly depend on the first step in the search phrase and the inner product of Bloom filter. Figure 10a describes the time consumption of search phrase changing with the size of file set. Our scheme and EliMFS grow slowly while CKSER linearly increases. The complexity of the search time in our scheme is O(log n) which is efficient than that of EliMFS. In Figure 11a,b, we set the number of files to be the same (*n* = 8000). Figure 11b demonstrates that the search time in CKSER do not changes as the number of keywords because they build index for each file. According to the above discussions, our scheme is efficient.

## 7. Conclusions

In this paper, we have proposed an efficient, auto correction retrieval scheme for data management in edge computing. The proposed scheme considers multi-keyword auto correction as well as the keyword weight of query keywords. We take an automatic error correction for the query keywords instead of similar words extension, which can tolerate spelling mistakes as well as reduce the complexity of index storage space. By the combination of TF-IDF value of keywords and the syntactic weight of query keywords, keywords who are more important will obtain higher relevance scores. We construct an R-tree index building with the encrypted keywords and the children nodes of which are the encrypted identifier FID and Bloom filter BF of files who contain this keyword. The secure index will be uploaded to the edge computing and the search phrase will be performed by the edge computing which is close to the data source. Then EDs sort the matching encrypted file identifier FID by relevance scores and upload them to the cloud server (CS). Performance analysis with actual data indicated that our scheme is efficient and accurate. In our future work, we will further discuss the possibility of enhancing the security in the energy internet system based on ensuring efficiency.

## Figures and Tables

**Table 1:** Notations and descriptions.

Symbol	Description
*f*	Plaintext file
*c*	Ciphertext file
$F=\{f_{1},f_{2},\ldots ,f_{n}\}$	The set of *n* plaintext files
$C=\{c_{1},c_{2},\;\ldots ,c_{n}\}$	The set of *n* ciphertext files
$W=\{w_{1},\;w_{2},\;\ldots ,\;w_{m}\}$	Keyword dictionary
*FID*	The encrypted identifier of files
KW(q)	The keyword weight.
*I*	The index of keyword dictionary
$Q^{\prime }$	Original query keywords
$Q=\{q_{1},q_{2},\ldots ,q_{k}\}$	Query keywords after auto correction
TQ	The trapdoor of the keywords *Q*
BFQ	The Bloom filter of *Q*

**Table 2:** An example of Dependency Relation.

Example	Types of Dependency Relation
Service, Attitude	Adjective modification relation: amod(NN,JJ)
Accept, Speed	Verb modification relation: advmod(VB,RB)
High, Quality	Noun topic modification relation: nsubj(JJ,NN)
Run, Fast	Adjective complement modification relation: comp(VB,JJ)

**Table 3:** Comparison of supported functions.

Schemes	MRSE	Wang’s	Fu’s	EliMFS	Our Scheme
Multi-keyword	√	√	√	√	√
Relevance ranking	√	√	√	√	√
Auto correction	×	×	×	×	√
Keyword weight	×	×	√	×	√
updating	×	×	√	×	√

(×: not supported; √: supported).
